# Combination of APACHE Scoring Systems with Adductor Pollicis Muscle Thickness for the Prediction of Mortality in Patients Who Spend More Than One Day in the Intensive Care Unit

**DOI:** 10.1155/2018/5490346

**Published:** 2018-06-03

**Authors:** Elahe Nematifard, Seyed Hossein Ardehali, Shaahin Shahbazi, Hassan Eini-Zinab, Zahra Vahdat Shariatpanahi

**Affiliations:** ^1^International Branch, Shahid Beheshti University of Medical Sciences, Tehran, Iran; ^2^Department of Anesthesiology and Critical Care, Shohada Tajrish Hospital, Shahid Beheshti University of Medical Sciences, Tehran, Iran; ^3^Department of Internal Medicine, Mostafa Khomaini Hospital, Ilam University of Medical Sciences, Ilam, Iran; ^4^Department of Community Nutrition, National Nutrition and Food Technology Research Institute, Faculty of Nutrition and Food Technology, Shahid Beheshti University of Medical Sciences, Tehran, Iran; ^5^Department of Clinical Nutrition, National Nutrition and Food Technology Research Institute, Faculty of Nutrition and Food Technology, Shahid Beheshti University of Medical Sciences, Tehran, Iran

## Abstract

**Background:**

The objective of the present study was to compare the ability of Acute Physiology and Chronic Health Evaluation (APACHE) scoring systems with the combination of an anthropometric variable score “adductor pollicis muscle (APM) thickness” to the APACHE systems in predicting mortality in the intensive care unit.

**Methods:**

A prospective observational study was conducted with the APM thickness in the dominant hand, and APACHE II and III scores were measured for each patient upon admission. Given scores for the APM thickness were added to APACHE score systems to make two composite scores of APACHE II-APM and APACHE III-APM. The accuracy of the two composite models and APACHE II and III systems in predicting mortality of patients was compared using the area under the ROC curve.

**Results:**

Three hundred and four patients with the mean age of 54.75 ± 18.28 years were studied, of which 96 (31.57%) patients died. Median (interquartile range) of APACHE II and III scores was 15 (12–20) and 47 (33–66), respectively. Median (interquartile range) of APM thickness was 15 (12–17) mm, respectively. The area under the ROC curves for the prediction of mortality was 0.771 (95% CI: 0.715–0.827), 0.802 (95% CI: 0.751–0.854), 0.851 (95% CI: 0.807–0.896), and 0.865 (95% CI: 0.822–0.908) for APACHE II, APACHE III, APACHE II-APM, and APACHE III-APM, respectively.

**Conclusion:**

Although improvements in the area under ROC curves were not statistically significant when the APM thickness added to the APACHE systems, but the numerical value added to AUCs are considerable.

## 1. Introduction

Predictive scoring systems have been developed to measure the severity of the disease and the prognosis of patients in the intensive care unit (ICU). Such measurements are helpful for clinical decision-making, standardizing research, and comparing the quality of patient care in the ICUs. The Acute Physiologic and Chronic Health Evaluation (APACHE) scoring system is one of the predictive scoring systems widely used in the world. The most frequently cited APACHE models are APACHE II and III; however, APACHE IV has also been validated. The APACHE II severity score is based upon the worst variables during the initial 24 hours in the ICU [1]. APACHE III resembles APACHE II, except that several variables have been added. APACHE III was developed in 1991 [2] and validated and further updated in 1998 [3], with incorporation of additional physiological measures, chronic health measures, and disease classification.

Malnutrition is a common problem in the ICU, and many patients admitted to the ICU have some degree of malnutrition. It has been shown that loss of lean body mass is associated with increased rate of morbidity and mortality in the intensive care setting [4]. There are many objective methods for measuring lean body mass in critically ill patients including biochemical indices, computed tomography, ultrasound imaging, dual-energy X-ray absorptiometry, bioelectrical impedance analysis, muscle circumferences, muscle thickness, and skinfold thickness. The accuracy, precision, specificity, and sensitivity of these measures vary significantly and have their own limitations [5–7]. The presence of edema and abnormal fluid shifts after the first day of admission decreases the precision of bioelectrical impedance analysis and anthropometric measurements [8]. Metabolic changes in critically ill patients alter serum biochemical markers such as albumin levels [8]. In individuals who are malnourished, the lean body mass decreases dramatically. Assessment of the thickness of adductor pollicis muscle (APM) could be a valid index in evaluating the muscle compartments of the body and therefore nutritional status. Among anthropometric measurements, measurement of APM thickness on the day of admission is a preferred method because it has a unique anatomic position with no covering of subcutaneous fat and can be directly measured by a fast, easy, low-cost, and noninvasive device. Evaluation of thicknesses of other muscles such as triceps requires subcutaneous fat measurement and a use of a formula for calculation [9]. Two studies have reported that the APM thickness on the day of admission predicts morbidity and mortality in critically ill patients [10, 11]. If APM thickness at the time of ICU admission is sensitive to predicting outcomes, then incorporating it directly into the predictive scoring models like APACHE II and III may increase the predictive value of these models. The purpose of this study was to determine whether a nutrition score on admission measured by APM thickness combined with APACHE II and APACHE III scores could increase prediction of mortality in the ICU.

## 2. Materials and Methods

**Figure 1:**
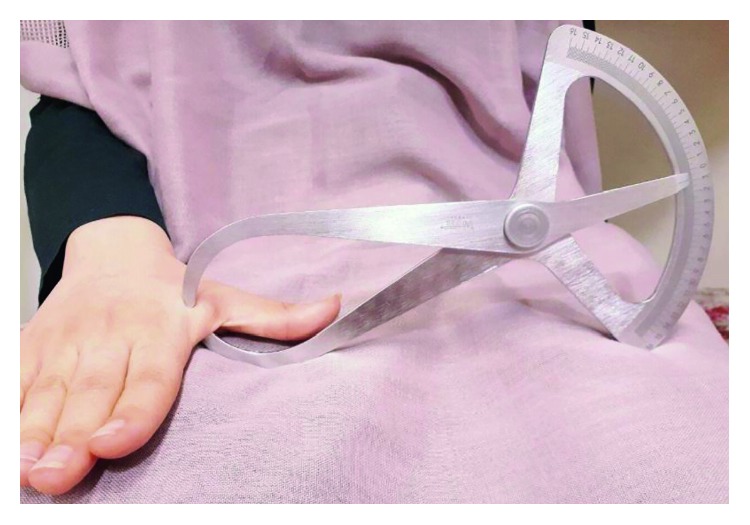
Measuring adductor pollicis muscle thickness.

**Figure 2:**
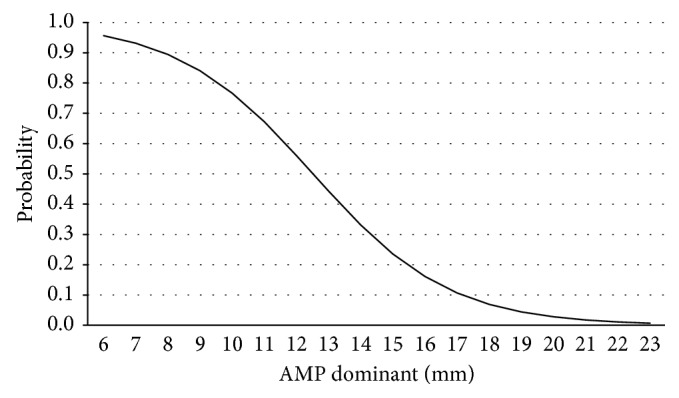
Probability of death by adductor pollicis muscle thickness.

This prospective study was conducted in a tertiary care university hospital between June 2014 and November 2016. The study protocol was approved by the responsible ethics committee, and informed consent was obtained from participants or their relatives. Patients were enrolled if they were older than 16 years. Anyone with splints, casts, neuromuscular weakness, and edema in the upper extremities were excluded from the study. Patients who had readmission episode were included only in their first admission. Furthermore, those transferred from other ICUs were excluded. The baseline medical history, physical examination, and APACHE II and III were recorded for all patients. All data were collected by a single physician. The APACHE II and III scores were gathered using the method presented by Knaus et al. [1, 3]. The APACHE II scores were calculated using 12 physiologic variables, age, and previous health status [1]. For APACHE III scores, the 18 acute physiological variables were used with the score ranging from 0 to 252, the age score from 0 to 24, and the chronic health evaluation from 0 to 23 [3]. The range of APACHE II and APACHE III scores was from 0 to 71 and from 0 to 299 points, respectively. The APACHE scores were calculated by the worst values taken during the first 24 hours after admission. APM thickness was measured from the dominant hand (as reported by the patient or their relative). APM thickness was assessed by Caliper (Vogel, Germany) in the emergency room or at the time of the ICU admission. It was measured while the patient's elbow was flexed at a 90 degree angle and the forearm was resting on the patient's torso. The caliper was applied across the APM situated in a triangle formed by the extended thumb and the index finger, with a 10 g/mm pressure (Figure 1). The average of three consecutive measurements was considered as a measure of the APM thickness for each individual [9]. To generate the composite APM and APACHE II score and give score for the APM variable, a multivariate logistic regression analysis between mortality and the APM thickness, with controlling 12 physiologic variables of APACHE II system, was performed. Results indicated that the coefficient (*B*) of the APM thickness was close to the coefficient of the Glasgow Coma Scale (GCS). Thus, the weighting scores for the APM thickness was derived from a procedure used in the GCS scores in APACHE II system so that the APM thickness lower than 15 mm subtracted from the score of 15 was the given APM score added to APACHE II scores. In patients with APM > 15 mm, the allocated score was zero. The reason for giving scores to muscle thicknesses lower than 15 mm is that, in this point, which is the median of the APM thickness, the probability of death is reduced based on the slope of the probability of the death curve showed in Figure 2. Hence, a composite model of APACHE II-APM was made with the range of 0 to 86 scores.

The primary outcome was in-hospital mortality, and all APM measurements were obtained by a single trained registered dietitian to minimize errors in measurement.

## 3. Statistical Analysis

The “Med Calculator” software was used to calculate the sample size. The calculation's underlying assumption was to increase the area under curve (AUC) of 0.874 for APACHE III mortality projection by more than 5 points with APACHE-APM (AUC = 0.93). All statistical analyses were performed with SPSS software version 21, and *p* values < 0.05 were considered significant. Patients' characteristics are reported as mean ± SD, median (interquartile range) for continuous variables, and in frequencies and percentages for categorical ones. Logistic regression analysis was performed to assess if the APM thickness on admission represents significant risk for mortality. To estimate weight for the APM thickness, multivariate logistic regression analysis was used to determine the relation between mortality and the APM thickness while controlling for other physiologic variables (12 physiologic variables for APACHE II and 18 physiologic variables for APACHE III). The area under the receiver-operating curves (ROC curve) to predict mortality was used to compare APACHE II, APACHE III, APACHE II-APM, and APACHE III-APM systems.

## 4. Results

**Figure 3:**
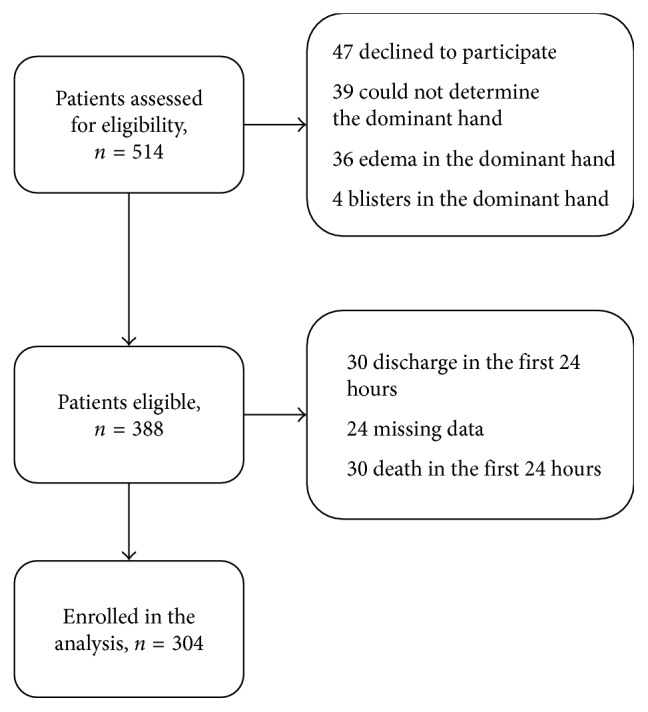
Flowchart of patients.

**Figure 4:**
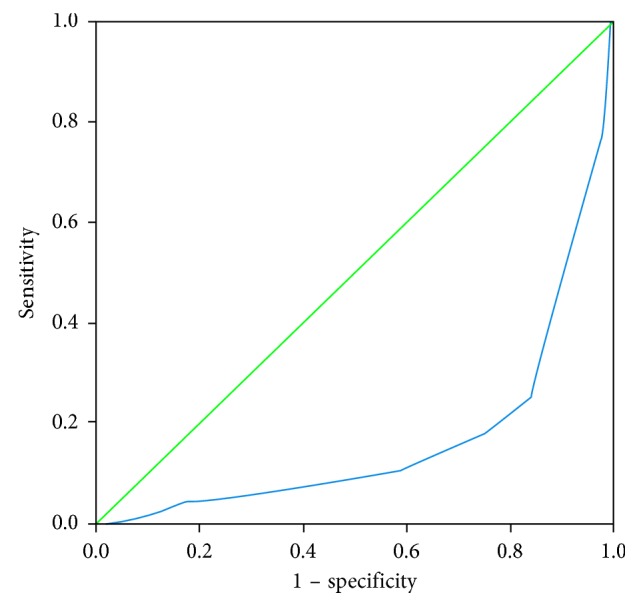
ROC curve for prediction of mortality using APM thickness. The area under the curve (AUC) is 0.166.

A total of 388 patients were consecutively enrolled in the study over the 29 months period. Eighty-four were excluded due to discharge or death before the second admission day and missing data. Therefore, 304 patients (117 females and 187 males) were included in the study (Figure 3).  Table 1 shows the baseline characteristics of the patients. Primary ICU diagnosis were cardiovascular or vascular disorder (*n*=34), respiratory disorder (*n*=49), gastrointestinal disorder (*n*=54), neurologic disorder (*n*=61), sepsis (*n*=21), trauma (*n*=46), orthopedic disorder (*n*=21), and gynecologic disorder (*n*=18). Ninety-six (31.5%) patients died. Median (interquartile range) of the APM thickness in patients who died was 13 (12–14) and in other patients was 16 (14–18). The minimum thickness of the APM was 6 millimeters (mm), and the maximum thickness was 21 mm. Logistic regression analysis showed that with each millimeter decrease in the APM thickness, risk of death is increased by 38% (Table 2). The area under the ROC curves for the prediction of mortality was 0.166 (95% CI: 0.115–0.217) for APM thickness (Figure 4).

Median (interquartile range) of the APACHE II score in patients who died and who survived was 20 (16–24) and 14 (11–17), respectively. The minimum and maximum score was 2 and 36, respectively. Logistic regression analysis showed that with each point increase in the APACHE II score, risk of death is increased by 20% (Table 2). This value for risk of death by the APACHE II-APM score is 28% (Table 2).

Median (interquartile range) of the APACHE III score in patients who died and who survived was 70 (51–86) and 41 (28–54), respectively. The minimum and maximum score was 5 and 146, respectively. Logistic regression analysis showed that with each point increase in the APACHE III score, risk of death is increased by 5.4% (Table 2). This value for risk of death by the APACHE III-APM score was 6.4% (Table 2).

To generate the composite APM and APACHE III score and give score for the APM variable, this variable was categorized by the probability of the death curve based on its thickness (Figure 2). APM thickness was categorized to three groups: APM thickness equal and less than 10 mm with the highest probability of death rate, APM thickness between 10 and 15 mm (median value) where the probability of death is reduced with a sharp slope, and APM thickness more than 15 mm (reference category) where the slope of the curve and probability of death is reduced. Demographic and outcome data between patients in each of the 3 APM groups are shown in Table 3. Logistic regression analysis was performed with death as dependent variable and categorized APM as covariate. Analysis showed that, in the category with APM thickness equal and less than 10 mm, risk of death is 40.7 times more than the reference category (OR = 40.7; 95% CI: 17.4–150.9; *p* < 0.001), and in the category with APM thickness between 10 and 15 mm, risk of death is 6.9 times more than the reference category (OR = 6.9; 95% CI: 4.6–19.6; *p* < 0.001). Thus, the weighting score for the APM thickness equal and less than 10 mm was 40 and the weighting score for the APM thickness between 10 and 15 mm was 7. Hence, a composite model of APACHE III-APM was made with the range of 0 to 339 scores.

The area under the ROC curves for the prediction of mortality was 0.771 (95% CI: 0.715–0.827), 802 (95% CI: 0.751–0.854), 0.851 (95% CI: 0.807–0.896), and 0.865 (95% CI: 0.822–0.908) for APACHE II, APACHE III, APACHE II-APM, and APACHE III-APM, respectively.

## 5. Discussion

Our study showed that the numerical values of AUC increased insignificantly in APACHE II-APM and APACHE III-APM scoring systems. Malnutrition that may be present on admission in ICU is associated with increased duration of hospitalization and mortality [12]. Many anthropometric variables could assay lean body mass and malnutrition. The reason for selecting the APM thickness as a malnutrition index is that its measurement is easy and fast and has a well-defined anatomical position [9]. Two studies have shown that the APM thickness is associated with mortality in the ICU [10, 11]. The first was conducted on 248 medical and surgical patients in a tertiary care ICU. They found that risk of death was 6.3 times more in patients with the APM thickness lower than 9 mm. Another study was conducted on 127 ICU patients, and it showed that risk of death was 3.8 times more in patients with low APM thickness. Our findings are similar to those of the two studies as we showed with each 1 mm decrease in APM thickness, risk of death is increased by 38%.

APACHE II-APM and APACHE III-APM scores appeared to increase the accuracy of mortality predictions that APACHE II or III scores alone. No other studies have assessed the power of adding anthropometric indices to APACHE scores, but two studies have evaluated a combination of the APACHE II score with the BMI in patients with acute pancreatitis, to predict severity of the disease with information in the first 24 hours of hospital admission. Johnson et al. [13] showed that combination of the APACHE II with BMI scores by simple addition improved categorical prediction of severity (mild or severe) in 186 patients with acute pancreatitis. Papachristou et al. [14] evaluated 102 acute pancreatitis patients to determine if the combination of the APACHE II and BMI adds any predictive value to the APACHE II score for severe outcomes. They concluded that although obesity increases the severity of acute pancreatitis, combination of the APACHE II and BMI scores on admission is not more accurate than APACHE II. In the critically ill patient, muscle mass is a better indicator of nutritional status than BMI [15]. Body mass index (BMI) is a measure of weight adjusted for height. Factors such as age, sex, ethnicity, and muscle mass can influence the BMI. The body is composed of fat and fat-free mass. Fat-free mass includes muscle, bones, and organs. BMI does not allow the determination of body composition. For example, some overweight (high BMI) men have normal fat mass and higher muscle mass.

As malnutrition that may be present on admission in ICU is associated with worse clinical outcomes [16], adding nutritional variables could improve the power of predictive models.

## 6. Limitations

This study has some limitations which have to be pointed out. First, sixty patients were excluded due to discharge or death before the second day of admission, as we were not able to get consent or calculate an APACHE score for them. Second, it was a single-center trial with a small sample size. Furthermore, the AUC for APM was poor. It should be noted that we do not expect the AUC for APM to have a large value. APM is a single variable like other variables in APACHE scoring systems.

## 7. Conclusion

As the new AUC values are greater than the APACHE system AUC values, it is suggested to consider nutritional parameters such as measurement of the APM thickness in the APACHE scoring systems in studies with larger sample size.

## Figures and Tables

**Table 1:** Comparison of variables between survivors and nonsurvivors.

	Survivors (*n*=208)	Nonsurvivors (*n*=96)	*p* value	Total (*n*=304)
Age^∗^	58.73 ± 17.2	52.90 ± 18.4	0.01	54.74 ± 18.28
Gender^∗∗^			1	
Male	125 (41.1%)	62 (20.4%)	0.52	187 (61.55)
Female	83 (27.3%)	34 (11.2%)		117 (38.4)
Serum albumin^∗^	3.3 ± 0.7	2.96 ± 0.6	0.01	3.19 ± 0.72
APACHE II^∗∗∗^	14 (11–17)	20 (16–24)	0.001	15 (12–20)
APACHE III^∗∗∗^	41 (28–54)	70 (51–86)	0.001	47 (33–66)
APMT (mm)^∗∗∗^	16 (14–18)	13 (12–14)	0.001	15 (12–17)
Subtype^∗∗^			0.04	
Medical	15 (4.9%)	13 (4.2%)		28 (9)
Surgical	89 (29.2%)	96 (31.5%)		185 (61)
Trauma	46 (15.1%)	45 (14.8%)		91 (30)

APACHE: Acute Physiology and Chronic Health Evaluation Score; APMT: adductor pollicis muscle thickness; ^∗^independent *t*-test (mean ± SD); ^∗∗^chi square (*n*, %); ^∗∗∗^Mann–Whitney (median, Q_1_–Q_3_).

**Table 2:** Logistic regression and AUR for mortality according to APM thickness and scoring systems.

Variable	OR	95% CI	*p* value	AUR
APM	0.623	0.553–0.701	<0.001	0.166
APACHE II	1.20	1.140–1.263	<0.001	0.771
APACHE II-APM	1.287	1.211–1.367	<0.001	0.851
APACHE III	1.054	1.039–1.068	<0.001	0.802
APACHE III-APM	1.064	1.049–1.080	<0.001	0.865

AUR: area under the Roc curve; APM: adductor pollicis muscle; APACHE: Acute Physiology and Chronic Health Evaluation.

**Table 3:** Demographic and outcome data between patients in each of the 3 APM groups.

	APMT ≤ 10 mm (*n*=30)	10 > APMT ≤ 15 (*n*=143)	APMT > 15 (*n*=131)	*p* value
Age^∗^	58.3 ± 15.5	56.5 ± 17.9	54.74 ± 18.28	0.1
Gender^∗∗^				0.001
Male	10 (3.3%)	77 (25.5%)	98 (32.5)	
Female	18 (6%)	66 (21.9%)	33 (10.9)	
Serum albumin (mg/dL)^∗^	2.90 ± 0.6	3.20 ± 0.7	3.24 ± 0.7	0.9
APACHE II^∗∗∗^	17 (13–20.75)	16 (12–20)	14 (12–20)	0.01
APACHE III^∗∗∗^	60 (45.25–75.25)	47 (35–70)	45 (31–59.75)	0.02
Survival^∗∗^	16 (14–18)	13 (12–14)	15 (12–17)	0.001
Alive	5 (17%)	80 (26.5%)	121 (40.1%)	
Death	23 (76.6%)	63 (44%)	10 (7.6%)	
Admission category^∗∗^				0.01
Medical	12 (3.9%)	4 (1.3%)	12 (3.9)	
Surgical	64 (21%)	49 (16.1%)	72 (23.6)	
Trauma	23 (7.5%)	41 (13.4%)	27 (8.8)	

APACHE: Acute Physiology and Chronic Health Evaluation Score; APMT: adductor pollicis muscle thickness; ^∗^ANOVA (mean ±  SD); ^∗∗^chi square (*n*, %); ^∗∗∗^Kruskal–Wallis.
